# MiR‐9‐enriched mesenchymal stem cells derived exosomes prevent cystitis‐induced bladder pain via suppressing TLR4/NLRP3 pathway in interstitial cystitis mice

**DOI:** 10.1002/iid3.1140

**Published:** 2024-02-02

**Authors:** Xiangrong Cui, Xingyu Bi, Xiuping Zhang, Zhiping Zhang, Qin Yan, Yanni Wang, Xia Huang, Xueqing Wu, Xuan Jing, Hongwei Wang

**Affiliations:** ^1^ Reproductive Medicine Center, The affiliated Children's Hospital of Shanxi Medical University, Children's Hospital of Shanxi Shanxi Maternal and Child Health Hospital Taiyuan China; ^2^ Clinical Laboratory Shanxi Provincial People's Hospital (Fifth Hospital) of Shanxi Medical University Taiyuan China; ^3^ Laboratory of Hematology Second Hospital of Shanxi Medical University Taiyuan China

**Keywords:** extracellular vesicle, interstitial cystitis/bladder pain syndrome, mesenchymal stem cell, miR‐9, NLRP3 inflammasome, TLR4

## Abstract

**Background:**

Inflammatory response of central nervous system is an important component mechanism in the bladder pain of interstitial cystitis/bladder pain syndrome (IC/BPS). Exosomes transfer with microRNAs (miRNA) from mesenchymal stem cell (MSCs) might inhibit inflammatory injury of the central nervous system. Herein, the purpose of our study was to explore the therapeutic effects by which extracellular vesicles （EVs） derived from miR‐9‐edreched MSCs in IC/BPS and further investigate the potential mechanism to attenuate neuroinflammation.

**Methods:**

On the basis of IC/BPS model, we used various techniques including bioinformatics, cell and molecular biology, and experimental zoology, to elucidate the role and molecular mechanism of TLR4 in regulating the activation of NLRP3 inflammasome in bladder pain of IC/BPS, and investigate the mechanism and feasibility of MSC‐EVs enriched with miR‐9 in the treatment of bladder pain of IC/BPS.

**Results:**

The inflammatory responses in systemic and central derived by TLR4 activation were closely related to the cystitis‐induced pelvic/bladder nociception in IC/BPS model. Intrathecal injection of miR‐9‐enreched MSCs derived exosomes were effective in the treatment of cystitis‐induced pelvic/bladder nociception by inhibiting TLR4/NF‑κb/NLRP3 signal pathway in central nervous system of IC/BPS mice.

**Conclusions:**

This study demonstrated that miR‐9‐enreched MSCs derived exosomes alleviate neuroinflammaiton and cystitis‐induced bladder pain by inhibiting TLR4/NF‑κb/NLRP3 signal pathway in interstitial cystitis mice, which is a promising strategy against cystitis‐induced bladder pain.

## Graphical abstract

This study demonstrated that extracellular vesicles derived from miR‐9‐enriched mesenchymal stem cells can freely cross the blood‐brain barrier into the spinal cord tissue, and then target TLR4 receptors on the activated spinal cord glial cell membrane of interstitial cystitis/bladder pain syndrome (IC/BPS) model to inhibit the activation of NLRP3 inflammatorome and release of inflammatory factors, which can reduce neuron stimulation and restore neuron firing threshold, so as to desensitize neurons and achieve the purpose of treating bladder pain in IC/BPS patients.

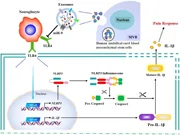

## 1 INTRODUCTION

Interstitial cystitis/bladder pain syndrome (IC/BPS) is a clinical syndrome of unclear precise etiology characterized by frequent and urgent urination, suprapubic and pelvic incapacitating pain after bladder filling, and relief after urination.^1^ According to the latest data, the global incidence of IC/BPS is 0.06% ~ 30% (male/female ratio is about 1:10), showing an increasing trend year by year.^2^ Bladder pain is a prominent symptom of IC/BPS, characterized by hyperalgesia, abnormal pain and spontaneous pain, and can even cause mental dysfunction such as depression, anxiety, panic, etc., seriously affecting patients' daily work ability and quality of life.^3^, ^4^, ^5^ However, at present, the treatment of IC/BPS bladder pain is difficult and ineffective, and there is no clear etiology and effective treatment.^6^ To explore the molecular mechanism of bladder pain, pain‐sensitizing targets and treatment methods in IC/BPS is of great practical significance for improving the symptoms of patients with bladder pain and further preventing the accompanying psychiatric diseases. Our previous studies have demonstrated that altered TLR4 activation plays a crucial role in bladder nociception independent of inflammation and urianry dysfunction in the IC/BPS model, providing clues for potential mechanisms and therapeutic targets of IC/BPS pain.^7^

The etiology of IC/BPS is complex, and the exact pathogenesis of IC/BPS is still unclear at present. There are a variety of etiological hypotheses, among which the main one is the theory of autoimmune damage.^8^ Toll‐like receptor (TLR) is an important pattern recognition receptor in innate immunity, which can initiate signal a cascade of responses to extracellular “danger” signals, and then trigger the release of immune cell pro‐inflammatory factors, such as tumor necrosis factor (TNF)‐α, interleukin (IL)‐6, IL‐1β, and so on.^9^ TLR4 is an important toll‐like receptor that recognizes pathogen‐related molecular patterns and injury‐related molecular patterns, and regulates innate or adaptive immune responses.^10^ TLR4 is expressed in a variety of cell types, including neurons and glial cells (microglia and astrocytes) in the central nervous system, immune cells (monocytes/macrophages) and nonimmune cells (bladder epithelial cells), which can mediate the production of pro‐inflammatory cytokines, resulting in enhanced central and peripheral immune signals, and further exacerbate nociceptive transmission, once activated.^11^, ^12^ Furthermore, recent studies have demonstrated that the activation of spinal cord glial cells and the release of inflammatory factors play an important role in the production and transmission of pain signals.^13^, ^14^ It has been shown that peripheral nerve injury could induce spinal cord neurogliocyte activation in chronic neuropathic pain models, which is closely related to TLR4‐mediated Nod‐like receptor protein 3 (NLRP3) inflammasome activation.^15^, ^16^ Thus, it is necessary to elucidate the relationship between the chronic pain in IC/BPS and NLRP3 inflammasome induced inflammatory cascade which triggered by the activation of TLR4.

Mesenchymal stem cells (MSCs) effectively alleviate neurological deficits and improve rehabilitation in patients with acute or chronic neurological diseases and injuries.^16^, ^17^ In addition, intrathecal delivery of bone marrow‐derived MSCs can inhibit spinal cord inflammation and pain caused by nerve injury.^18^ However, MSCs engrafting to central nervous system (CNS) tissues may cause capillary embolism, and genetic material variation after repeated subculture in vitro.^18^ Furthermore, our previous studies confirmed that MSCs had tumor‐like transformation risk when cocultured with glioma cells directly and indirectly in vitro.^19^, ^20^

At present, it is widely accepted that the beneficial effects of MSCs are mainly attributable to their paracrine factors, such as cytokines, growth factors, and EVs, rather than cell differentiation and replacement of damaged cells.^21^ In addition, EVs released by MSCs are currently recognized as an indispensable part of the cell microenvironment and are considered to play a major role in signal delivery.^22^ As a promising cell‐free therapeutic tool, MSCs‐derived EVs can simulate almost all the biological characteristics of MSCs, such as mediation of inflammatory response, tissue damage, and regeneration.^23^, ^24^ These EVs are membrane vesicles with 30‐100 nanometers, which transfer specific sets of functional RNAs (mRNAs and noncoding RNAs) and proteins to recipient cells.^25^, ^26^, ^27^ In light of existing research, miRNAs secreted from cells within EVs can be transferred to other cell compartments which essentially function as efficient therapeutic molecules for neuropathic pain.^28^ Identifying specific miRNAs and their roles related to neuroinflammation and chronic pain would give us better understanding about the mechanisms of IC/BPS therapeutic action, facilitating the development of cell‐free therapeutic strategy for IC/BPS therapy.

Based on the above evidence, in present study, we have focused on the neuroinflammation of IC/BPS, and explored whether miR‐9‐enriched MSCs derived exosomes alleviated neuroinflammaiton and cystitis‐induced bladder pain by inhibiting TLR4/NLRP3 pathway in interstitial cystitis mice, aiming to provide a new avenue for IC/BPS therapy.

## 2 METHODS

### 2.1 Cystitis induction

All experiments were approved by the Institutional Animal Care and Use Committee at the Shanxi Medical University. Sixty female C57BL/6 mice (22–26 g in weight) were used due to a higher incidence of IC/BPS in females than males in humans and the easier feasibility of intravesical surgery in females.^29^, ^30^ Mice were anesthetized by intraperitoneal injection with 0.3 ~ 0.4 mL of 2% pentobarbital sodium solution. The bladder was then catheterized urethrally with a polyethylene catheter (PE‐50), instilled with 1 mL of protamine (10 mg/mL; Sigma‐Aldrich), and retained for 45 min. After draining the instilled liquid, 1 mL of LPS (750 μg/mL; Solarbio; L8880) was instilled and kept for 30 min. Repeat the preceding steps for three consecutive days. At day 14 after cystitis induction, mice were analyzed for phenotypic and functional changes or treated with MSC‐EVs or TAK‐242 followed by phenotypic and functional analyses.

### 2.2 Bladder histology

Bladders were harvested and fixed overnight with standard formalin in 4% paraformaldehyde (PFA), embedded in paraffin for microscopic evaluation by hematoxylin and eosin (H&E) staining. During histological evaluation, bladder inflammation was scored in based on leukocyte infiltration in the lamina propria and mucosal edema as previously described (1+: mild infiltration with no or mild edema, 2 + : moderate infiltration with moderate edema, and 3+: moderate to severe infiltration with severe edema).^7^

### 2.3 Spinal cord immunofluorescence

L6–S1 spinal cord was harvested and processed for formalin fixation in 4% PFA and dehydrated with 30% surcrose at 4°C. Tissue specimens were cut into 20 μm sections. Sections were blocked with 5% bovine serum albumin for 1 h at 20–25°C and then incubated against the following primary antibodies: NeuN (ab177487, 1:200; Abcam), GFAP (ab7260, 1:300; Abcam) and IBA1 (ab178846, 1:100; Abcam). Then sections were exposed to FITC‐conjugated secondary antibodies (ab150077, 1:100; Abcam), and mounted with DAPI, and the images were acquired with a Leica DFC350 FX microscope. Image J software was performed to quantify the fluorescent intensity of each image.

### 2.4 Splenocyte cytokine production

Splenocytes were isolated from mice spleens and seeded in 48‐well plates at a density of 2 × 10^6^ cells in 1 ml per well,^7^ which resuspended in RPMI‐1640 medium supplemented with 10% FBS (Millipore), and treated with LPS (Solarbio; L8880) at 10‐fold escalating dosages ranging from 10^−5^ to 10^2^ μg/mL for 24 h. In short, the relative levels of IL‐1, IL‐6, and TNF‐α in conditioned culture supernatants were analyzed by ELISAs using commercial kits (Sigma‐Aldrich) according to the manufacturer's instructions.

### 2.5 Cytotoxicity assay

To measure cytotoxicity, released lactate dehydrogenase (LDH) activity in cell culture supernatants was assessed by an LDH cytotoxicity assay kit (KGT02424, KeyGEN BioTECH) in accordance with the manufacturer's instructions.

### 2.6 Bladder nociception

Urinary bladder distention‐evoked visceromotor response (VMR) method was performed to detect bladder nociception as described previously.^31^ Birefly, mice were anesthetized with isoflurane by mask and allowed to ventilate spontaneously. The bladder was catheterized via the urethra with a PE‐50 catheter. After the electrode implantation into the superior oblique musculature of the abdomen and the chest inferior to the heart, mice were placed inside the plexiglass restraining tubes for 30 min for acclimation. For the measurement of VMR to urinary bladder distension (UBD), graded pressure (10, 20, 30, 40, 50, 60, 70, and 80 mmHg) of airflow with a pressure controlled device was injected into the bladder. The electromyographic (EMG) signals were amplified using the amplifier (A‐M System; model 1700; Carlsborg). Data were recorded real‐time using CED Spike 2 software (CED Micro1401, Cambridge Electronic Design).

### 2.7 Pelvic pain analysis

Pelvic pain was detected by quantifying referred withdrawal threshold of the suprapubic region in the response to applied force with von Frey filaments (Aesthesio) as described previous.^7^ Before testing, mice were acclimatized to the Plexiglas chambers (6 × 10 × 12 cm) environment for 20 min. Five a series of von Frey flaments (Stoelting, Wood Dale, IL) with different forces (0.04, 0.16, 0.4, 1, and 4 g) were used perpendicularly to the skin of suprapubin region for 6–8 s with an interval of 5 min repeated 10 times. A positive response was defined as immediately licking or grabbing the stimulated area, or jumping. Response frequency was calculated as the percentage of positive responses to each filament.

### 2.8 Voiding habit analysis

Mice were placed in individual metabolic cages (Columbus Instruments) for 24 h real‐time time recording of voiding habits (voiding frequency and voided volume per micturition) as described previously.^7^ During the recording process, the mice were allowed free access to drinking water but were restrained from solid food to prevent feces from interfering with measurement of urine volume. The voiding frequency was recorded for 24 h with 2 min intervals using Oxymax software (Columbus Instruments).

### 2.9 Cell transfection

Lipofectamine 2000 transfection reagent was used fo rthe transient transfection of NC‐mimic and miR‐9 mimic into MSCs (1.0 × 10^5^ cells/well) according to the manufacturer's instructions.

### 2.10 MSC‐EVs isolation and identification

MSC‐EVs were extracted from the culture supernatants of human umbilical cord derived MSCs obtained from Chongqing Stem Cell Therapy Engineering and Technology Center. The 24 h before the collection of EVs from MSCs transfected with miR‐9, the medium was replaced with medium containing 10% EV‐depleted FBS. In brief, EVs isolation was carried out by differential ultracentrifugation as previously described.^32^ The cell supernatants were collected and centrifuged successively at 300×*g* (10 min, 4°C), 2000×*g* (10 min, 4°C), and 10,000×*g* (30 min, 4°C) to remove dead cells and large cell debris. Then the supernatants were collected and ultracentrifuged at 100,000×*g* (70 min, 4°C), followed by being washed in PBS and ultracentrifuged at 100,000×*g* (70 min, 4°C) to remove contaminating proteins. The morphology of purified EVs were observed by transmission electron microscopy (TEM) (JEM‐1200EX, JEOL), Nano‐flow cytometry, and EVs markers.

### 2.11 Intrathecal injection

Intrathecal injection of MSC‐EVs in mice was performed as previous described protocol.^33^ Under isoflurane inhalation anesthesia, a microsyringe was punctured vertically into spinal canal in the intervertebral space between L5 and L6 spinous process (the lowest lumbar spinous process). The formation of an “S” shape by the tail known as tail‐fick reaction indicated a successful puncture, the syringe angle was reduced to 30°, and then MSC‐EVs (5 μg in 25 μL) or PBS was injected. After injection, the needle remained at the puncture site for more than 15 s to ensure reagent delivery and avoid leakage.

### 2.12 Western blot analysis

L6–S1 spinal cord was harvested and processed for extracting total protein using RIPA lysis buffer containing proteinase and phosphatase inhibitor cocktail solution (KeyGEN BioTECH). Total protein was extracted from spinal cord tissues, microglia, and EVs. Protein concentration was quantified using the BCA Protein Assay Kit (Sigma‐Aldrich; Merck KGaA). Total protein (30 μg/lysates) was loaded on a 10% SDS‐PAGE. Proteins were separated and transferred onto PVDF membranes (Merck Millipore). Membranes were incubated in 5% bovine serum albumin (BSA) for 1 h at room temperature followed by primary antibodies including TLR4 (1:1000; CST), p65 NF‐κb (1:1000; CST), p‐p65 NF‐κb (1:1000; CST), Hsp70 (1:1000; CST), CD63 (1:1000; CST), NLRP3 (1:1000; Abcam), Caspase‐1 (1:1000; Abcam), IL‐1β (1:1000; ABclonal), IL‐18 (1:1000; Abcam)，GAPDH (1:5000; Abcam) and β‐actin (1:5000; Abcam) at 4°C for overnight. Membranes were washed with Tris‐buffered saline/0.1% Tween (TBST) three times, incubated with secondary antibodies conjugated with HRP for 1.5 h. Protein bands were probed using an enhanced chemiluminescence kit (Millipore) and an Image J software (Bio‐Rad).

### 2.13 Fluorescence quantitative PCR

Total RNA was isolated from mice L6–S1 spinal cord and microglia using TRIzol (Termo Fisher). cDNA was generated using reverse transcription kit (Takara). FQ‐PCR was performed using SYBR Premix Ex Taq kit (Bao Biological Engineering, Dalian, China) with a CFX‐96 (BIO‐RAD). We evaluated samples for gene expression of IL‐1β, IL‐6, TNF‐α, TLR4, miR‐9 (Table 1). All experimental samples were determined in triplicate and averaged. To calculate the relative expression of miR‐9 and mRNA, the 2^−ΔΔCt^ method was used.

**Table 1:** Sequences of primers used for fluorescence quantitative PCR in this study.

Gene	Primer sequence (forward)	Primer sequence (reverse)
TLR4	ATGGCATGGCTTACACCACC	GAGGCCAATTTTGTCTCCACA
miR‐9	GCCCGCTCTTTGGTTATCTAG	CCAGTGCAGGGTCCGAGGT
IL‐6T	CTGCAAGAGACTTCCATCCAG	AGTGGTATAGACAGGTCTGTTGG
IL‐1β	CGAAGACTACAGTTCTGCCATT	GACGTTTCAGAGGTTCTCAGAG
TNF‐α	CTGAACTTCGGGGTGATCGG	GGCTTGTCACTCGAATTTTGAGA
GAPDH	AGGTCGGTGTGAACGGATTTG	TGTAGACCATGTAGTTGAGGTCA
β‐actin	AACGCTTCACGAATTTGCGT	GCTCCAACCGACTGCTGTCACCTTC
U6	CTCGCTTCGGCAGCACA	AACGCTTCACGAATTTGCGT

Abbreviations: GAPDH, glyceraldehyde 3‐phosphate dehydrogenase; IL, interleukin; NLRP3, NLR family pyrin domain containing 3; TLR4, toll‐like receptor 4; TNF‐α, tumor necrosis factor‐α.

### 2.14 Bioinformatic analysis

The Gene Expression Omnibus (GEO, https://www.ncbi.nlm.nih.gov/geo/) from the National Center for Biotechnology Information was performed, and data from the GSE60033 were downloaded for miRNA expression analysis. The expression of microRNAs involved in chronic neuropathic pain was calculated using R software (R × 64 3.3.3). The microRNA Data Integration Portal (miRDIP, http://ophid.utoronto.ca/mirDIP/) was used for target gene prediction.

### 2.15 Statistical analysis

Results were analyzed using IBM SPSS Statistics 23.0 (IBM Corporation) and are expressed as means ± standard error of the mean. Data were compared using a Student's *t*‐test for two groups or one‐way analysis of variance followed by a Student‐Newman‐Keuls post hoc test for multiple groups. *p*‐value of .05 was considered statistically significant.

## 3 RESULTS

### 3.1 Bladder nociception is associated with increased systemic and central TLR4‐mediated inflammatory responses in IC/BPS

**Figure 1:**
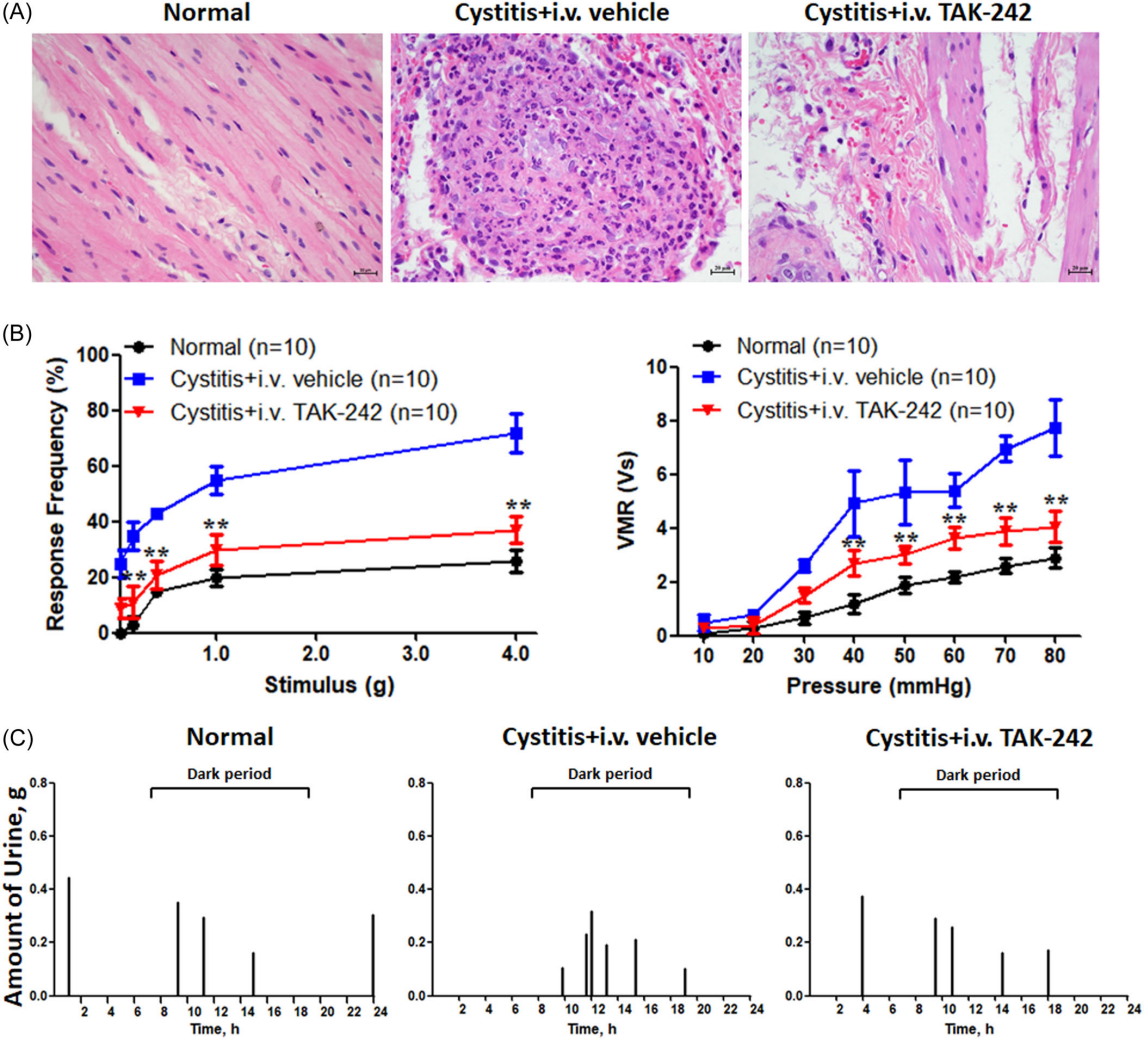
TLR4 play a critical role in bladder nociception of IC/BPS mice. (A) H&E assays were conducted to observe the effect of TAK‐242 on IC/BPS‐induced bladder inflammation (scale bar, 100 μm, ×200). (B) TAK‐242‐treated IC/BPS mice exhibited significantly alleviated pelvic mechanical allodynia to von Frey filament stimulation and bladder distention‐evoked VMRs compared with vehicle‐treated IC/BPS mice. (C) TAK‐242 treatment significantly increased mean volume voided per micturition, maximum volume voided per micturition, alleviating the number of voids of IC/BPS mice. Compared with the cystitis + i.v. vehicle group. *p < .05; **p < .01, *n* = 10.

To investigate the role of TLR4 in cystitis induced neuroinfammation and mechanical abnormal pain, we treated cystitis induced mice with a TLR4‐selective antagonist TAK‐242, and evaluated whether the inhibition of TLR4 activation could lead to reduction in pelvic and bladder nociceptive responses in the animal model. H&E assays were conducted to observe the effect of TAK‐242 on IC/BPS‐induced bladder inflammation. Compared to control group (*n* = 6; score: 0 ‐ +), the TAK‐242 group (score: 3+ for four bladder and 2+ for two bladders) and vehicle‐treated mice (score: 3+ for all six bladders) developed similar bladder inflammation (Figure 1A). Compared with vehicle‐treated mice, TAK‐242‐treated group exhibited significantly alleviated pelvic mechanical allodynia to von Frey filament stimulation and bladder distention‐evoked VMRs (Figure 1B). Additionally, TAK‐242 treatment also significantly increased mean volume voided per micturition, maximum volume voided per micturition, alleviating the number of voids of IC/BPS mice (Figure 1C). It was indicated that TLR4 plays a critical role in bladder nociception and voiding dysfunction independent of bladder inflammation in IC/BPS model.

### 3.2 Bladder nociception is associated with increased systemic and central TLR4‐mediated inflammatory responses in IC/BPS

**Figure 2:**
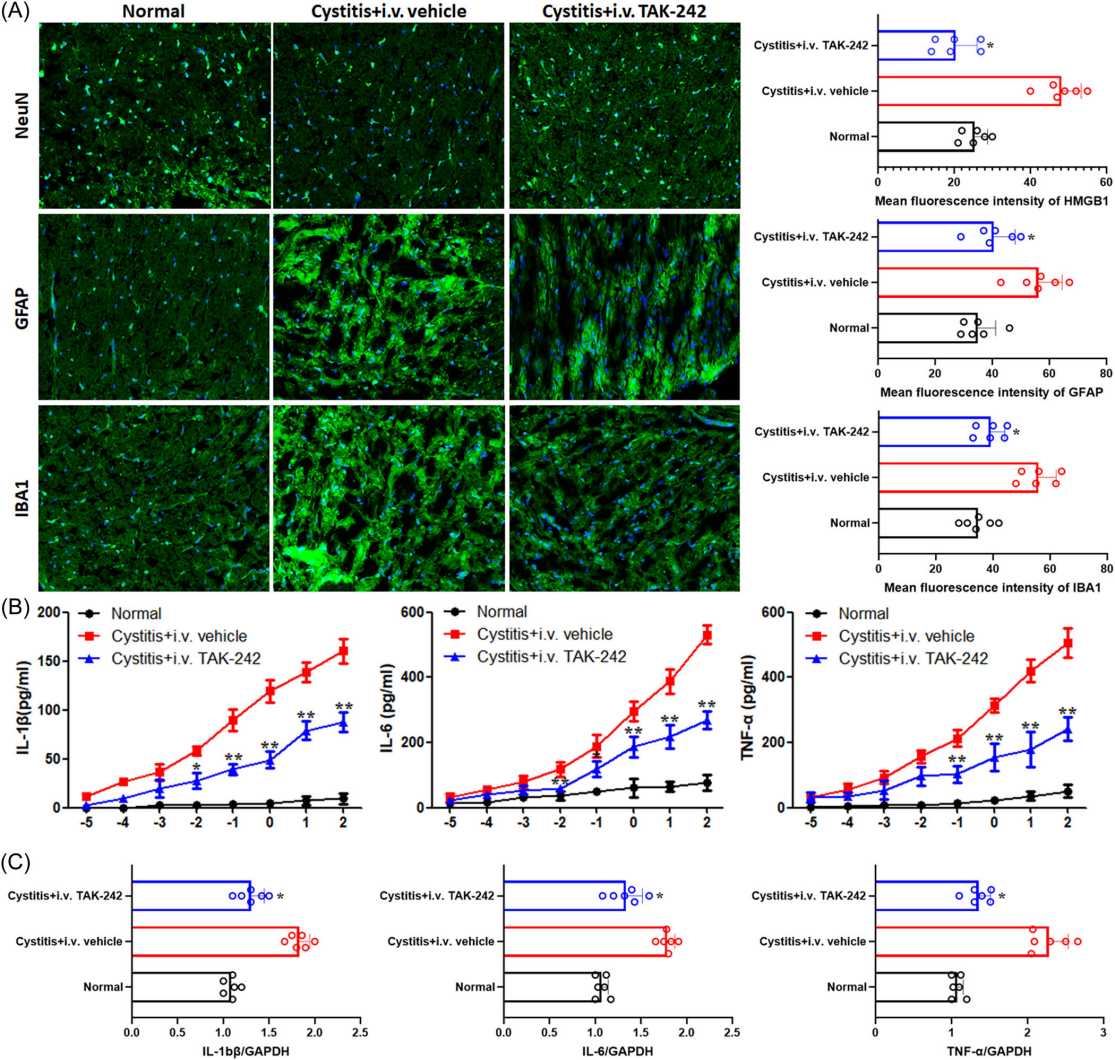
Bladder nociception is associated with increased systemic and central TLR4‐mediated inflammatory responses in IC/BPS. (A) Immunofluorescence analysis showing that expression levels of HMGB1, GFAP and IBA1 were significantly increased in lumbar spinal cords of IC/BPS mice compared with normal mice, and TAK‐242 treatment significantly decreased expression levels of HMGB1, GFAP and IBA1 in lumbar spinal cords of IC/BPS mice (scale bar, 100 μm, ×200). (B) ELASA analysis showing that splenocytes from TAK‐242‐treated IC mice produced significantly reduced levels of pro‐inflammatory cytokines in response to in vitro stimulation with LPS at dosages of 10^‐5^, 10^‐4^, 10^‐3^, 10^‐2^, 10^‐1^, 10^0^, 10^1^, 10^2^ g/mL compared with splenocytes from vehicle‐treated IC/BPS mice. (C) PCR analysis showing that lumbar spinal cords from TAK‐242‐treated IC/BPS mice expressed substantially reduced levels of mRNAs for pro‐inflammatory cytokines IL‐1, IL‐6, and TNF‐α compared with lumbar spinal cords from vehicle‐treated IC/BPS mice. Compared with the Cystitis+i.v. vehicle group. *p < .05; **p < .01, *n* = 6.

We conducted an immunofluorescence analysis to measure the expression of endogenous TLR4 ligand HMGB1, and glial activation markers GFAP and IBA1 in lumbar spinal cords, and found that HMGB1, GFAP and IBA1 were downregulated after TAK‐242 injection in the group with cystitis (Figure 2A). In addition, we conducted a ELASA assay to evaluate the expression of pro‐inflammatory cytokines IL‐1, IL‐6, and TNF‐α. In parallel to bladder nociception, splenocytes from TAK‐242‐treated IC mice produced significantly reduced levels of pro‐inflammatory cytokines in response to in vitro stimulation with LPS at dosages of 10^−5^, 10^−4^, 10^−3^, 10^−2^, 10^−1^, 10°, 10^1^, 10^2^ g/mL compared with splenocytes from vehicle‐treated IC/BPS mice (Figure 2B). Furthermore, lumbar spinal cords from TAK‐242‐treated IC/BPS mice expressed substantially reduced levels of mRNAs for pro‐inflammatory cytokines IL‐1, IL‐6, and TNF‐α compared with lumbar spinal cords from vehicle‐treated IC/BPS mice (Figure 2C). These observations indicate that the inflammatory responses in systemic (splenocyte) and central (spinal glial) derived by TLR4 activation is closely related to the cystitis‐induced pelvic/bladder nociception and frequent micturition in IC/BPS model.

### 3.3 MiR‑9 was identifed as a potential functional candidate effector for inhibiting TLR4

**Figure 3:**
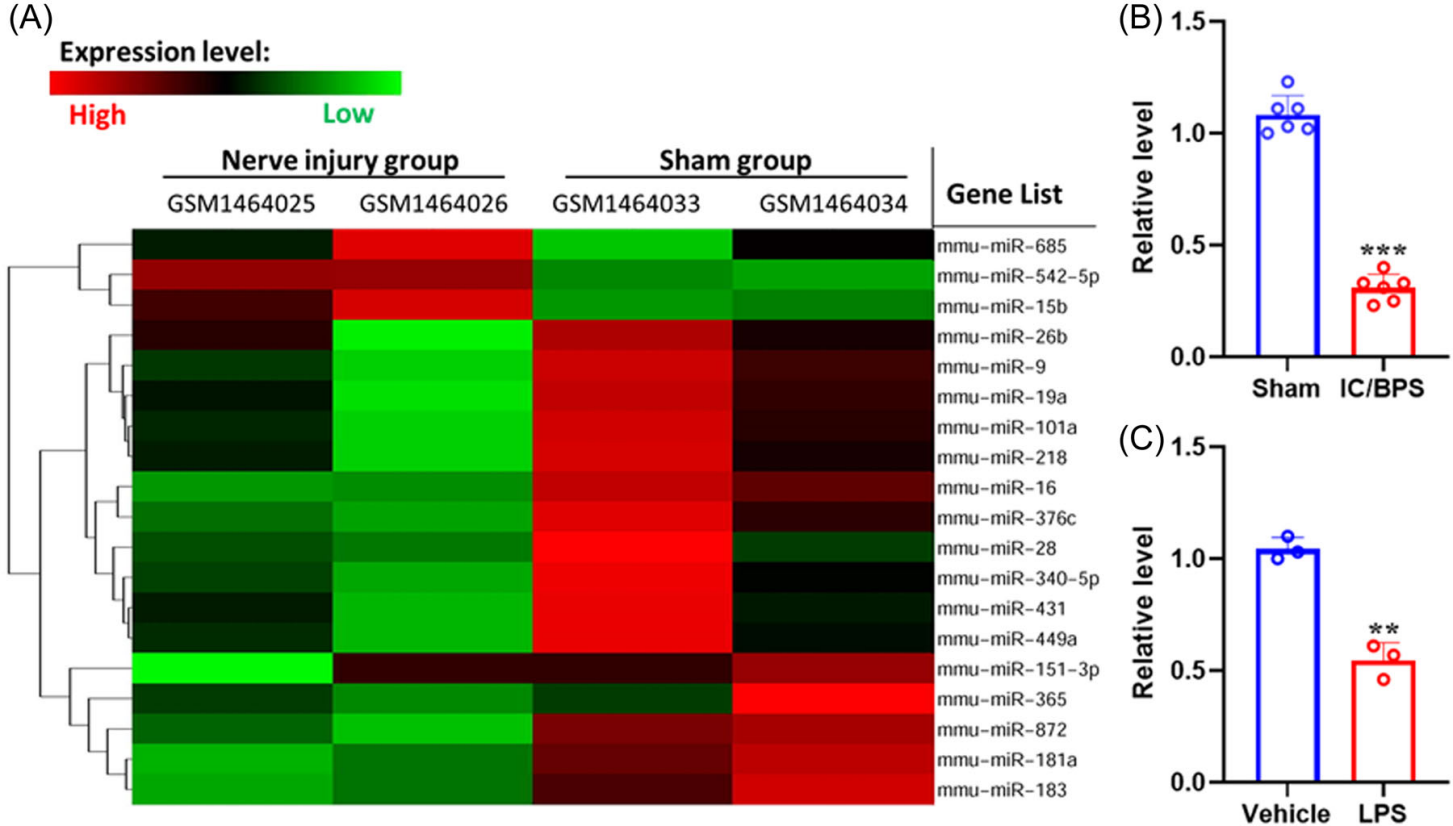
Prediction of pain‐related microRNAs by bioinformatics. (A) GEO database found that the expression of miR‐9 targeting TLR4 was decreased after nerve injury compared with the sham operation group. (B, C) The expression of miR‐9 was significantly decreased in the lumbar spinal cord tissue of the IC/BPS‐induced mice and in the primary astrocytes treated with LPS. Compared with sham or vehicle group. *p < .05; **p < .01, *n* = 3–6.

In recent years, related studies have shown that miRNA is involved in a variety of chronic pain diseases such as inflammatory myalgia and peripheral neuralgia, and miRNA expression changes significantly before and after onset. GEO database was used to obtain microRNAs expression data involved in neuropathic pain following a peripheral nerve injury, which found that the expression of miR‐9 targeting TLR4 was decreased after nerve injury compared with the sham operation group (Figure 3A). Combined with IC/BPS model, we found that the expression of miR‐9 was significantly decreased in the lumbar spinal cord tissue of the IC/BPS‐induced mice and in the primary astrocytes treated with LPS (Figure 3B,C).

**Figure 4:**
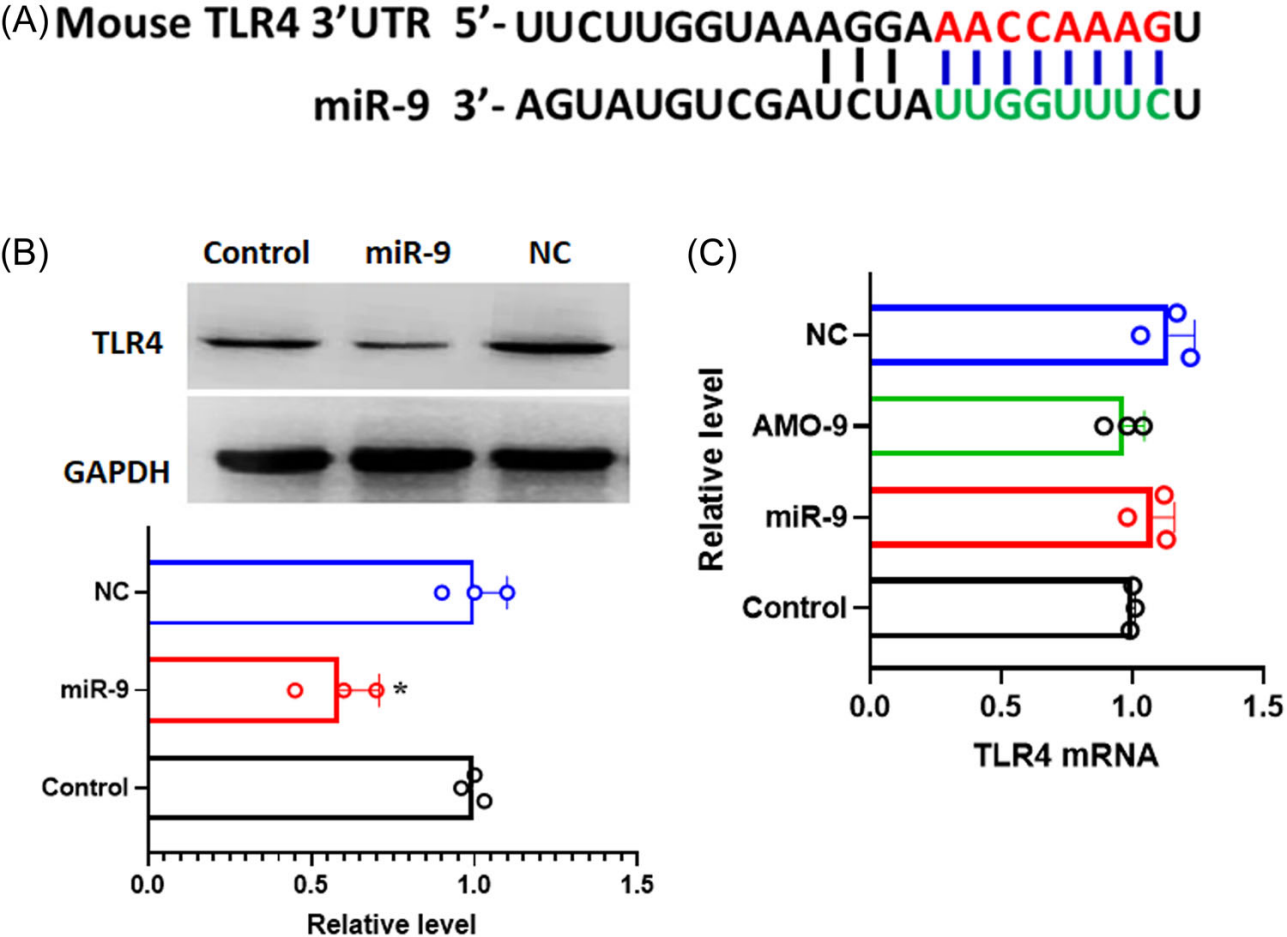
MiR‑9 was identifed as a potential functional candidate effector for inhibiting TLR4. (A) The putative binding site of miR‐9 to the 3′ UTR of TLR4 was predicted using the TargetScan and Starbase v2.0. (B and C) Overexpression of miR‐9 in astrocytes significantly inhibited TLR4 protein expression, but had no significant effect on mRNA levels. Compared with the NC group. *p < .05; **p < .01, *n* = 3.

Therefore, we subsequently investigated whether TLR4 was direct target of miR‐9. The putative binding site of miR‐9 to the 3′ UTR of TLR4 was predicted using the TargetScan and Starbase v2.0 (Figure 4A). Overexpression of miR‐9 in astrocytes significantly inhibited TLR4 protein expression (Figure 4B), but had no significant effect on mRNA levels (Figure 4C). Therefore, we speculate that chronic pain in IC/BPS mice may be caused by the downregulation of miR‐9 expression in glial cells, which leads to the activation of TLR4.

### 3.4 Characterization of MSC‐EVs

**Figure 5:**
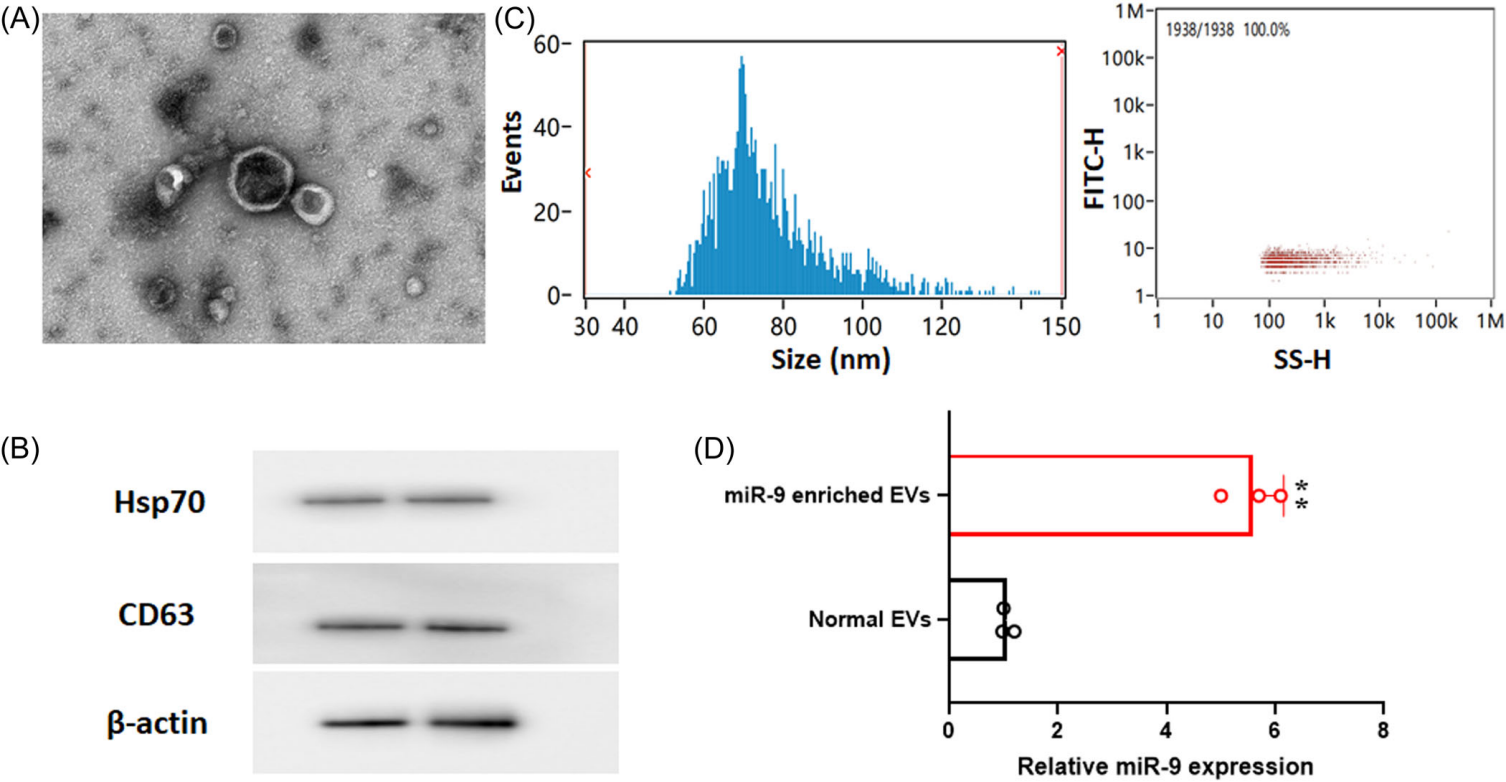
Characterization of MSC‐EVs. (A) The isolated EVs showed goblet vesicle‐like structures of different sizes under transmission electron microscopy. (B) Western blot analysis showing that EV protein markers (Hsp70 and CD63) were enriched in MSC‐EVs. (C) Nano‐flow cytometry (NanoFCM) results show showed that HUC‐MSCs‐EV was mainly distributed in the vicinity of 60–120 nm. (D) QRT‐PCR revealed that miR‐9 expression was increased in miR‐9 enriched EVs by approximately 6.20‐fold compared with normal EVs. Compared with the normal EVs group. *p < .05, *n* = 3.

To explore the therapeutic effects of EVs derived from miR‐9‐modified mesenchymal stem cells on IC/BPS, we first isolated MSC‐EVs from the culture supernatants of MSCs, and transmission electron microscopy (TEM), nanoparticles tracking analysis (NTA) by high sensitivity flow cytometry and western blot analysis were used to identify the obtained EVs. Images acquired by TEM showed isolated EVs as circular double‐layered vesicles with the common exosomal markers (Hsp70 and CD63) (Figure 5A,B). By Nano‐flow cytometry (NanoFCM) results, we further measured the accurate size of vesicles and sample concentration, which were approximate 30–150 nm in diameter and 2.39ere^10^ particles/mL (Figure 5C). Then, we compared the exosomal miR‐9 expression with qRT‐PCR, and revealed that miR‐9 expression was increased in miR‐9 enriched EVs by approximately 6.20‐fold compared with normal EVs (Figure 5D).

### 3.5 MiR‐9 enriched EVs alleviate the bladder nociception and frequent micturition in IC/BPS mice

**Figure 6:**
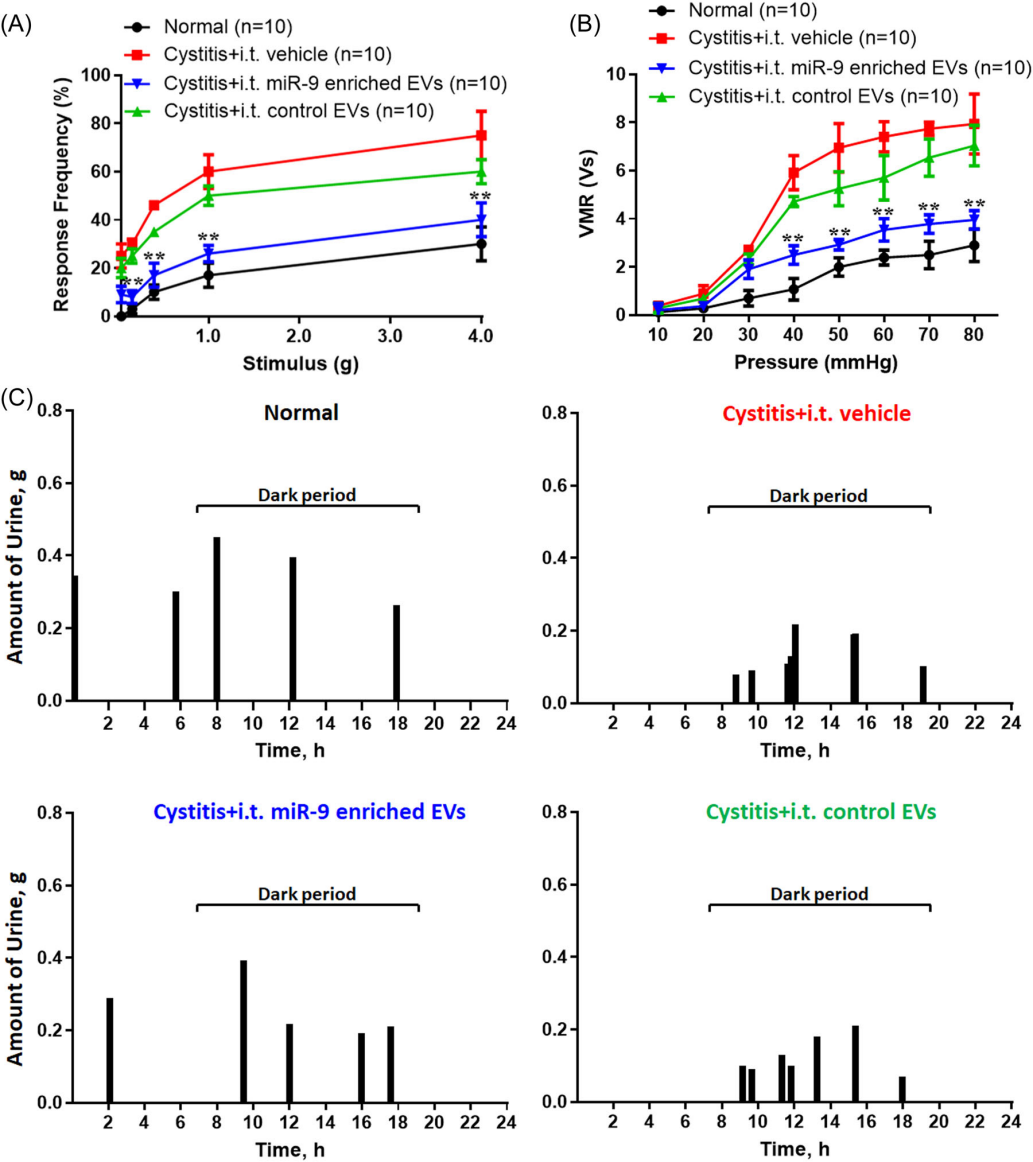
MSC‐EVs alleviate the bladder nociception and frequent micturition in IC/BPS mice. (A and B) miR‐9 enriched EVs‐treated IC/BPS mice exhibited significantly reduced pelvic nociceptive responses to von Frey filament stimulation and bladder distention‐evoked VMRs compared with vehicle‐treated IC/BPS mice. (C) miR‐9 enriched EVs‐treated IC/BPS mice also significantly increased mean volume voided per micturition, maximum volume voided per micturition, alleviating the number of voids of IC/BPS mice. Compared with the cystitis+i.t. vehicle group. *p < .05, *n* = 10.

IC/BPS mice were intrathecally treated with vehicle or EVs derived from miR‐9‐modified mesenchymal stem cells at day 14 after cystitis induction and evaluated for pelvic and bladder nociceptive responses and voiding habits 1 h after treatment. Compared with vehicle‐treated IC/BPS mice, miR‐9 enriched EVs‐treated IC/BPS mice exhibited significantly reduced pelvic nociceptive responses to von Frey filament stimulation and bladder distention‐evoked VMRs (Figure 6A,B). In addition, miR‐9 enriched EVs‐treated IC/BPS mice also significantly increased mean volume voided per micturition, maximum volume voided per micturition, alleviating the number of voids of IC/BPS mice (Figure 6C).

### 3.6 MiR‐9 enriched EVs attenuates the activation of glial cells and reduced systemic and central TLR4‐mediated inflammation in IC/BPS mice

**Figure 7:**
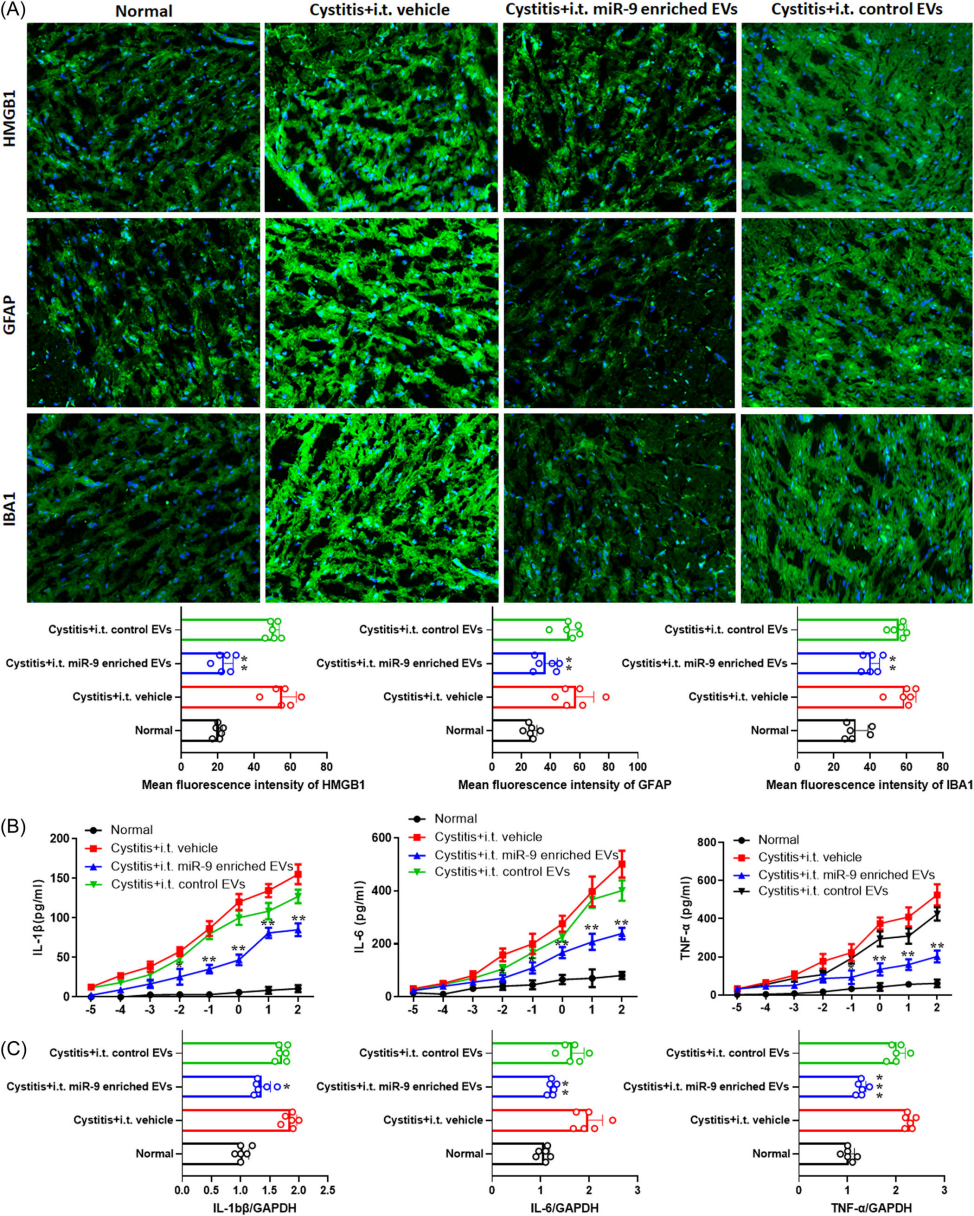
MiR‐9 enriched EVs attenuates the activation of glial cells and reduced systemic and central TLR4‐mediated inflammation in IC/BPS mice. (A) Lumbar spinal cords from miR‐9 enriched EVs‐treated IC/BPS mice expressed substantially reduced levels of HMGB1, GFAP and IBA1 compared with lumbar spinal cords from vehicle‐treated IC/BPS mice (scale bar, 100μm, ×200). (B) Splenocytes from miR‐9 enriched EVs‐treated IC/BPS mice produced significantly reduced levels of pro‐inflammatory cytokines IL‐1β, IL‐6, and TNF‐α in response to in vitro stimulation with LPS compared with splenocytes from vehicle‐treated IC/BPS mice. (C) QRT‐PCR analysis showed that expression levels of IL‐1β, IL‐6, and TNF‐α were also reduced significantly. Compared with the cystitis+i.t. vehicle group. *p < .05; **p < .01; ***p < .001, *n* = 6.

In parallel to pelvic/bladder nociceptive response attenuation, lumbar spinal cords from miR‐9 enriched EVs‐treated IC/BPS mice expressed substantially reduced levels of HMGB1, GFAP and IBA1 compared with lumbar spinal cords from vehicle‐treated IC/BPS mice (Figure 7A). In addition, splenocytes from miR‐9 enriched EVs‐treated IC/BPS mice produced significantly reduced levels of pro‐inflammatory cytokines IL‐1β, IL‐6, and TNF‐α in response to in vitro stimulation with LPS at a dosage range from 10^−5^ to 10^2^ μg/mL compared with splenocytes from vehicle‐treated IC/BPS mice (Figure 7B). Furthermore, consistent with the inhibition of pro‐inflammatory cytokines in systemic inflammation, mRNA levels of IL‐1β, IL‐6, and TNF‐α were also reduced significantly (Figure 7C), suggesting that EVs derived from miR‐9‐modified mesenchymal stem cells could attenuate the inflammatory responses in systemic and central derived by TLR4 activation in IC/BPS model.

### 3.7 MiR‐9 enriched EVs suppress the activity of NLRP3 inflammasome by inhibiting the TLR4/NF‑κb signal pathway in SDH of IC/BPS mice

**Figure 8:**
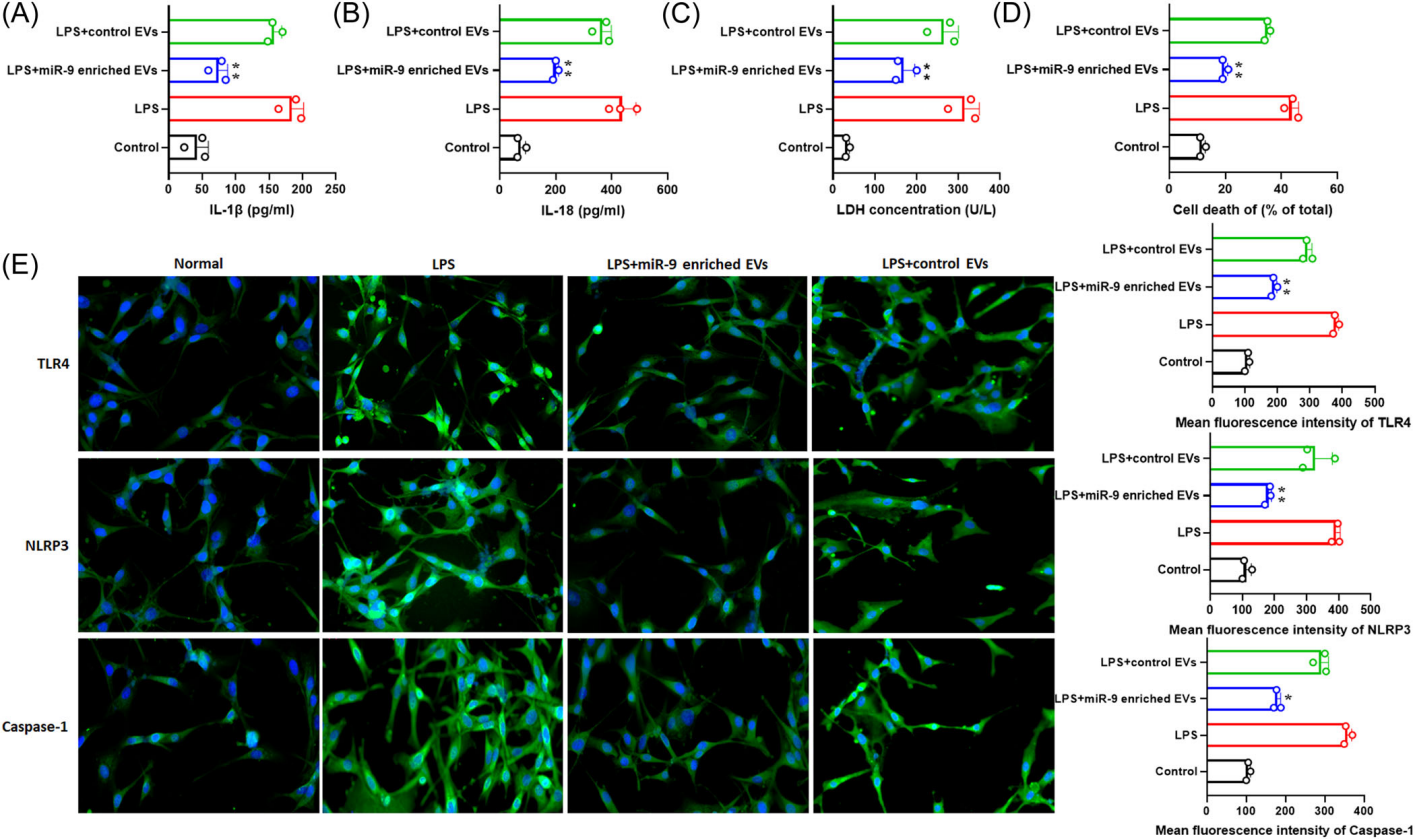
MiR‐9 enriched EVs suppress the activity of NLRP3 inflammasome by inhibiting the TLR4 signal pathway in micaroglia treated by LPS. (A–D) MiR‐9 enriched EVs significantly suppressed the LPS‐induced upregulation of pro‐inflammatory cytokines, including IL‐1β, IL‐18, LDH, and the rate of cell death compared with the LPS group. (E) Immunofluorescence analysis showed that miR‐9 enriched EVs also significantly reduced LPS‐induced upregulation of TLR4, NLRP3, and caspase‐1 (scale bar, 100μm, ×200). Compared with the LPS group. *p < .05; **p < .01, *n* = 3.

**Figure 9:**
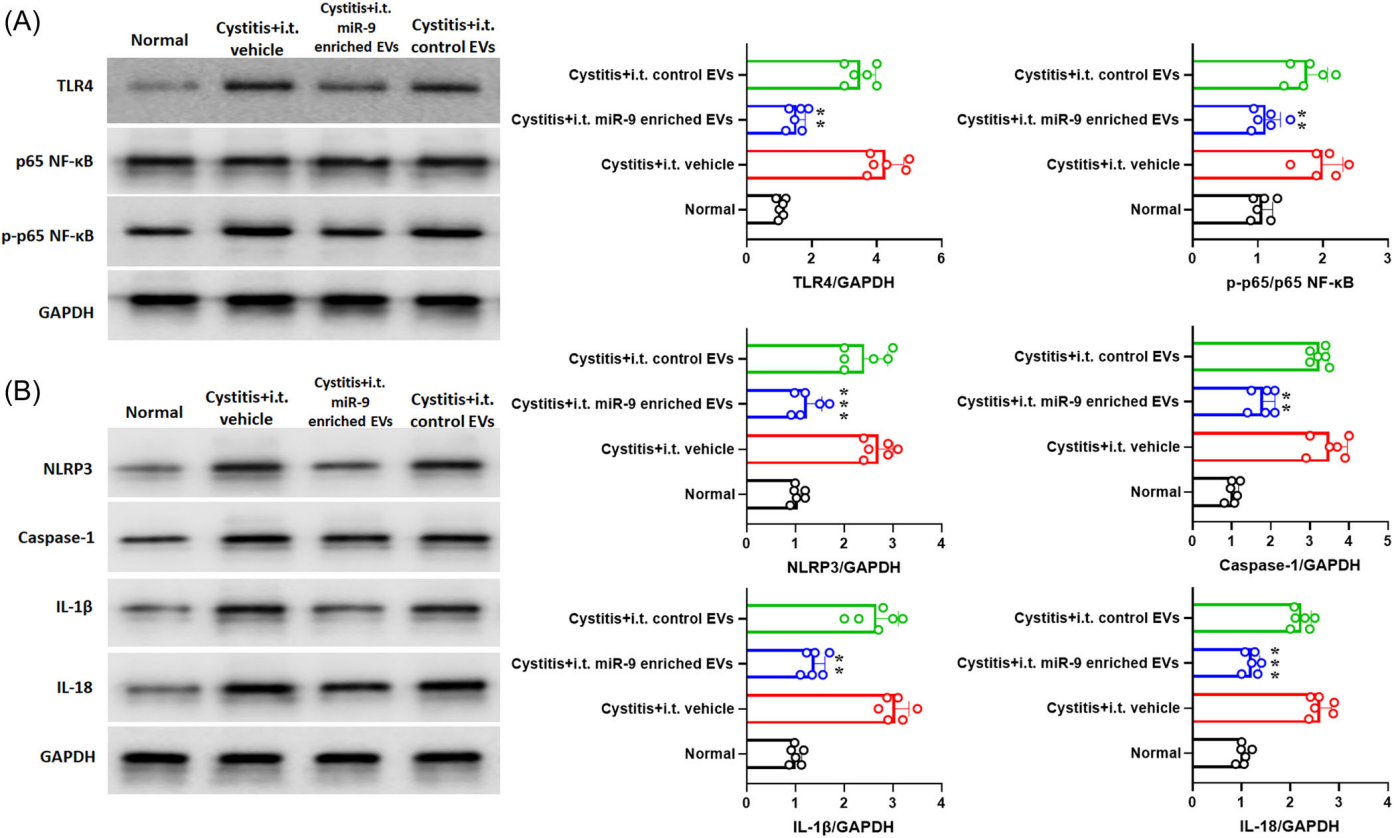
MiR‐9 enriched EVs suppress the activity of NLRP3 inflammasome by inhibiting the TLR4/NF‑κb signal pathway in SDH of IC/BPS mice. (A, B) Western blot analysis revealed that expression level of TLR4, phosphorylation ratio of NF‐κB (p65), NLRP3, caspase‐1, IL‐1β and IL‐18 were signifcantly increased in SDH of IC mice compared with normal mice, and intrathecal injection of miR‐9 enriched EVs signifcantly decreased expression level of all proteins in SDH of IC mice. Compared with the Cystitis+i.t. vehicle group. *p < .05; **p < .01, *n* = 6.

To further investigate the underlying mechanism that exosomal miR‐9 derived from MSCs attenuates TLR4‐mediated neuroinflammation in IC mice, miR‐9 enriched EVs were added into micaroglia treated by LPS. As expected, compared with the LPS group, miR‐9 enriched EVs significantly suppressed the LPS‐induced upregulation of pro‐inflammatory cytokines, including IL‐1β, IL‐18, LDH, and the rate of cell death (Figure 8). Moreover, immunofluorescence analysis showed that miR‐9 enriched EVs also significantly reduced LPS‐induced upregulation of TLR4, NLRP3, and caspase‐1 (Figure 8). Then, we further investigate whether exosomal miR‐9 derived from MSCs could attenuate the bladder nociception by inhibiting the TLR4/NF‐κb/NLRP3 signaling pathway induced inflammatory in IC mice. As shown in Figure 9A, western blot analysis revealed that compared with normal group, the expression level of TLR4 and phosphorylation ratio of NF‐κb (p65) were significantly increased in vehicle‐treated IC/BPS mice, and miR‐9 enriched EVs‐treated IC/BPS mice exhibited significantly reduced both of them. Western blot results showed the expression levels of NLRP3, caspase‐1, IL‐1β and IL‐18 were significantly inhibited in miR‐9 enriched EVs‐treated IC/BPS mice compared with vehicle‐treated IC/BPS mice (Figure 9B). These observations indicate that exosomal miR‐9 derived from MSCs suppress the activity of NLRP3 inflammasome, might by inhibiting the TLR4/NF‑κb signal pathway in SDH of IC/BPS mice.

## 4 DISCUSSION

**Figure 10:**
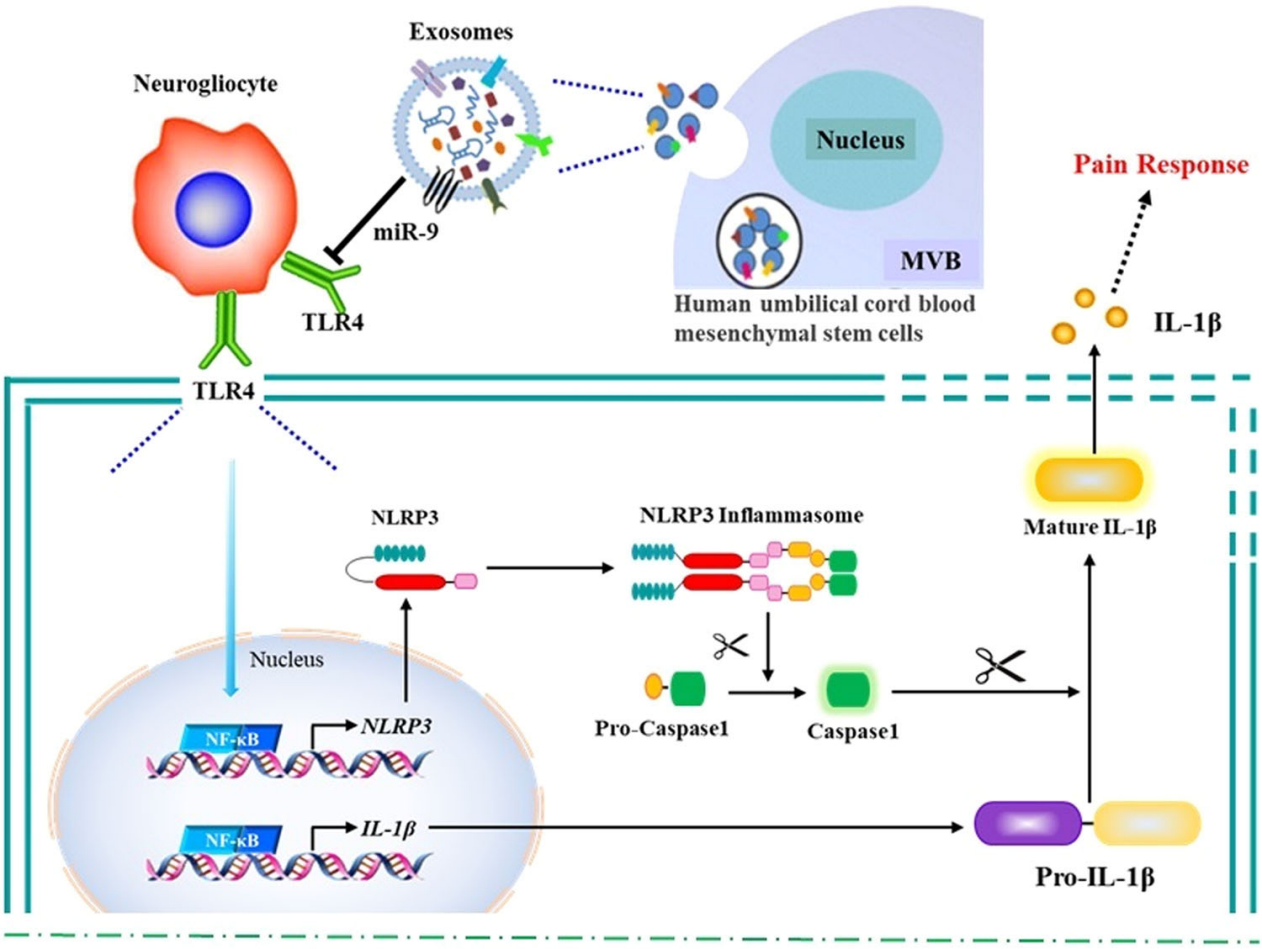
EVs derived from miR‐9‐modified mesenchymal stem cells could alleviate neuroinflammation and cystitis‐induced bladder pain by inhibiting TLR4/NLRP3 pathway in interstitial cystitis mice.

In the present study, we demonstrated that TLR4‐mediated neuroinflammation played a critical role in bladder nociception of IC/BPS model, and inhibition of TLR4 activation using TAK‐242 alleviated the spinal neuroinflammation and bladder nociception. In addition, miR‐9 was identified as potential candidate effector for inhibiting TLR4, and we assessed for the first time the therapeutic efficacy of exosomal miR‐9 derived from MSCs in IC/BPS. We found that miR‐9 enriched EVs treatment could alleviated the bladder nociception, frequent micturition, microgliosis, as well as systemic and central TLR4‐mediated inflammation in IC/BPS mice. Finally, we proved that EVs derived from miR‐9‐modified mesenchymal stem cells could alleviate neuroinflammation and cystitis‐induced bladder pain by inhibiting TLR4/NLRP3 pathway in interstitial cystitis mice (Figure 10).

IC/BPS is one of the most intractable diseases in urology today.^34^ As a prominent symptom of IC/BPS, bladder pain seriously affects patients' ability to perform daily work and quality of life.^35^ Due to the etiology and pathogenesis of IC/BPS remains elusive, traditional treatment methods do not guarantee a satisfactory therapeutic effect and even produce detrimental side effects.^36^ TLR4 activation has been found to induce and maintain a high intensity pain state in a variety of chronic pain disorders. Xing et al. have confirmed that the TLR4/NF‐κB pathway activated in the bottom tissue of the foot and dorsal root ganglion induces the release of pro‐inflammatory factors, which is an important factor causing postoperative pain in patients.^37^ It has been reported that the peripheral blood monocytes of IC/BPS patients can induce the release of pro‐inflammatory cytokines IL‐1β and IL‐6 under the stimulation of TLR4 agonist Lipopolysaccharide (LPS) in vitro, which is closely related to the severity of pain in patients.^37^ We have previously verified that TLR4‐deficient URO‐OVA mice developed significantly reduced bladder nociceptive responses, although similar bladder inflammation and voiding dysfunction, after cystitis induction.^7^ These positive results indicated that altered TLR4 activation plays a critical role in bladder nociception, providing a potential mechanistic insight and therapeutic target for IC/BPS pain.

To explore the therapeutic pathway of IC/BPS, whether through the central system or peripheral blood, we explored the role of glial cells in the central nervous system during this process. TLR4 is expressed in a variety of cell types, including neurons and glial cells (such as microglia and astrocytes) in the central nervous system, immune cells and nonimmune cells.^12^ Once activated, TLR4 could mediate the production of pro‐inflammatory cytokines, leading to increased central and peripheral immune signaling, which in turn enhances nociceptive transmission.^12^ In the meanwhile, recent studies have found that the activation of spinal cord glial cells and the release of inflammatory factors play a critical role in the production and transmission of pain signals.^13^ As two of the most important types of glia in the central nervous system, microglia and astroglia are mainly involved in pain perception and central regulation, and play an important role in the formation and maintenance of chronic pain, as well as the key factors in inflammatory response.^38^, ^39^ Activated pro‐inflammatory cytokines (such as IL‐1, IL‐6, TNF‐α) can regulate the synthesis and release of inflammatory factors by directly acting on the surface receptors of neurons or other immune cells, and sensitizing or reducing the neuronal firing threshold, leading to a long‐term amplification, diffusion, and migration of pain signals.^40^ Our previous studies have detected aberrant activation of microglia and astrocytes in the SDH of IC/BPS mice.^7^ In the present study, we found that TAK‐242 significantly decreased the expression levels of endogenous TLR4 ligand HMGB1, and glial activation markers GFAP and IBA1 in lumbar spinal cords, which meant the activation of spinal microglia and astrocytes plays an important role in chronic pain in IC/BPS mice and is closely related to TLR4‐mediated inflammatory response. Given small molecule inhibitors like TAK‐242 are limited in clinical practice due to their side effects. Therefore, to explore a safe and effective therapeutic tool applicable to clinical practice becomes the key to solve the problem.

Researchers have found that miRNA is involved in various chronic pain diseases such as inflammatory myalgia and peripheral neuropathy, and there are significant changes in miRNA expression before and after the onset of the disease.^41^, ^42^, ^43^, ^44^, ^45^ We obtained miRNAs expression data related to neuropathic pain after peripheral nerve injury using the GEO database, and found that miR‐9 expression targeting TLR4 decreased after nerve injury compared to the sham operation group. Furthermore, we found that the expression of miR‐9 was significantly decreased in the lumbar spinal cord tissue of the IC/BPS‐induced mice and in the primary astrocytes treated with LPS. Bazzoni et al. have confirmed that miR‐9 can prevent excessive inflammatory response and promote immune autostability and immune regulation in the inflammation induced by TLR4 activation.^46^ This indicated that chronic pain in IC/BPS mice may be caused by the downregulation of miR‐9 expression in glial cells, which leads to activation of TLR4. MiRNA is an excellent tool for gene therapy due to its simple structure, easy synthesis and modification, and strong predictability of targets. These findings suggest that miR‐9 can be used to inhibit the activation of TLR4 and its downstream downstream signaling pathway in the treatment of IC/BPS bladder pain.

In recent years, it is well‐known that exogenous stem cells, especially mesenchymal stem cells, have shown extensive application prospects as cell‐based therapies in preclinical and clinical studies. It has been reported that intrathecal delivery of bone marrow‐derived MSCs can inhibit spinal cord inflammation and pain caused by nerve injury.^18^ Further studies have shown that MSCs mainly participate in intercellular communication through EVs to exert biological functions such as immune regulation, transportation, and nutrition, alleviate tissue damage, and promote tissue repair.^22^ In addition, MSCs‐derived EVs can simulate almost all the biological characteristics of MSCs, regulate inflammatory responses, and promote tissue regeneration and damage repair.^23^ In the present study, we demonstrated that exosomal miR‐9 derived from MSCs exhibited favorable therapeutic efficacy in IC/BPS mice through inflammation modulation. As nano‐sized particles, EVs can freely penetrate the vascular wall and blood‐brain barrier, and their biological activities and functional properties are not reprogrammed by environmental influences, so their application safety and targeting are much higher than MSCs.^23^, ^24^ Therefore, EVs derived from MSCs, as a carrier of information exchange between cells, are expected to be a new therapeutic method in regenerative medicine.

To better understand the underlying mechanism that exosomal miR‐9 derived from MSCs attenuates TLR4‐mediated neuroinflammation in IC/BPS mice, we explored the role of inflammasome during this process. Peripheral nerve injury has been shown to induce spinal cord neurogliocyte activation in chronic neuropathic pain models and is closely related to TLR4‐mediated NLRP3 inflammasome activation.^15^, ^16^ In this study, we found that miR‐9 enriched EVs the effect of alleviating the bladder nociception similar to TAK‐242 IC/BPS mice. After added miR‐9 enriched EVs into microglia which treated by LPS, the IL‐1β, IL‐18, and LDH in medium, and the TLR4, NLRP3, and caspase‐1 in microglia were reduced, suggesting that miR‐9 enriched EVs alleviated microglia activation by inhibiting the activation of TLR4/NLRP3 pathway. Moreover, we further found that the expression levels of TLR4, NF‐κb (p65), NLPR3, caspase‐1, IL‐1β and IL‐18 in IC/BPS mice were significantly inhibited after intrathecal injection of miR‐9 enriched EVs, suggesting that exosomal miR‐9 derived from MSCs suppress the activity of NLRP3 inflammasome, might by inhibiting the TLR4/NF‑κb signal pathway in SDH of IC mice.

## 5 CONCLUSION

Taken together, this study demonstrated that EVs derived from miR‐9‐enriched MSCs can freely cross the blood‐brain barrier into the spinal cord tissue, and then target TLR4 receptors on the activated spinal cord glial cell membrane of IC/BPS model to inhibit the activation of NLRP3 inflammatorome and release of inflammatory factors, which can reduce neuron stimulation and restore neuron firing threshold, so as to desensitize neurons and achieve the purpose of treating bladder pain in IC/BPS patients.

## AUTHOR CONTRIBUTIONS

**Xingyu Bi**: Methodology; resources. **Zhiping Zhang**: Data curation; software. **Qin Yan**: Data curation; resources. **Yanni Wang**: Methodology; writing—original draft. **Xia Huang**: Conceptualization; resources. **Xueqing Wu**: Formal analysis; resources. **Hongwei Wang**: Formal analysis; funding acquisition; investigation; supervision; validation; visualization; writing—review and editing.

## CONFLICT OF INTEREST STATEMENT

The authors declare that they have no competing interests.

## ETHICS STATEMENT

This work was carried out under the project entitled “Mechanisms of exosomes derived from HUC‐MSCs enriched with miR‐9 regulating the activity of NLRP3 inflammasome through TLR4 in the treatment of bladder pain of interstitial cystitis/bladder pain syndrome”. This study was approved by the Institutional Animal Care and Use Committee of Shanxi Medical University (Ethics Ref. No. IRB‐GKY‐2020‐008), dated March 31, 2020. All experimental procedures were performed in accordance with the Ethical Guidelines from the International Council for Laboratory Animal Science.

## Data Availability

The datasets used and/or analyzed during the current study are available from the corresponding author on reasonable request.
